# Transcriptome Analysis of *Pistacia vera* Inflorescence Buds in Bearing and Non-Bearing Shoots Reveals the Molecular Mechanism Causing Premature Flower Bud Abscission

**DOI:** 10.3390/genes11080851

**Published:** 2020-07-25

**Authors:** Jubina Benny, Francesco Paolo Marra, Antonio Giovino, Bipin Balan, Tiziano Caruso, Federico Martinelli, Annalisa Marchese

**Affiliations:** 1 Department of Agricultural, Food and Forest Sciences, University of Palermo, Viale delle Scienze—Ed. 4, 90128 Palermo, Italy; jubina.benny@unipa.it (J.B.); bipin.balan@unipa.it (B.B.); tiziano.caruso@unipa.it (T.C.); 2 Department of Architecture (DARCH), University of Palermo, Viale delle Scienze—Ed. 8, 90128 Palermo, Italy; 3 Council for Agricultural Research and Economics (CREA), Research Centre for Plant Protection and Certification (CREA-DC), 90011 Bagheria, Italy; antonio.giovino@crea.gov.it; 4 Department of Plant Sciences, University of California, Davis, CA 95616, USA; 5 Department of Biology, University of Florence, Sesto Fiorentino, 50019 Florence, Italy; federico.martinelli@unifi.it

**Keywords:** *Pistacia vera*, polyamines, flower bud abscission, alternate bearing

## Abstract

The alteration of heavy (“ON/bearing”) and light (“OFF/non-bearing”) yield in pistachio (*Pistacia vera* L.) has been reported to result from the abscission of inflorescence buds on high yielding trees during the summer, but the regulatory mechanisms involved in this bud abscission remain unclear. The analysis provides insights into the transcript changes between inflorescence buds on bearing and non-bearing shoots, that we indicated as “ON” and “OFF”, and shed light on the molecular mechanisms causing premature inflorescence bud abscission in the pistachio cultivar “Bianca” which can be related to the alternate bearing behavior. In this study, a transcriptome analysis was performed in inflorescence buds of “ON” and “OFF” shoots. A total of 14,330 differentially expressed genes (DEGs), most of which are involved in sugar metabolism, plant hormone pathways, secondary metabolism and oxidative stress pathway, were identified. Our results shed light on the molecular mechanisms underlying inflorescence bud abscission in pistachio and we proposed a hypothetical model behind the molecular mechanism causing this abscission in “ON” shoots. Results highlighted how changes in genes expressed in nutrient pathways (carbohydrates and mineral elements) in pistachio “ON” vs. “OFF” inflorescence buds triggers a cascade of events involving trehalose-6-phosphate and target of rapamycin (TOR) signaling, SnRK1 complex, hormones, polyamines and ROS which end, through programmed cell death and autophagy phenomena, with the abscission of inflorescence buds. This is the first study reporting gene expression profiling of the fate of “ON” and “OFF” inflorescence buds associated with the alternate bearing in the pistachio.

## 1. Introduction

Pistachio (*P. vera* L.) originates in the arid areas of central Asia, in the areas of the Caspian Sea (Iran) and the territories between Afghanistan and Kyrgyzstan [1]. The pistachio is a wind-pollinated deciduous, dioecious tree which presents cyclic variation of fruiting, usually of two years, in which heavy production occurs during the “ON” year and less/no production in the following “OFF” year. The mechanism regulating the alternate bearing phenomenon in pistachio is unique [1,2]. In a mature pistachio tree, all the main vegetative and reproductive phases are carried out by the plant in a short period, between the mid of March and the end of May. The growth pattern of the current season’s shoot is exclusively dominant, and it extends from the vegetative terminal bud of the previous season’s shoot. Under each of the compound leaf on the current season’s growth, there is a single axillary bud. Most of these axillary buds differentiate into inflorescence primordia; therefore, flowering and fruit production occurs on 1-year-old wood [2,3,4].

Thus, unlike other alternate bearing crop species, pistachio produces floral buds on current-year shoot but, in the “ON” year, inflorescence buds start to detach starting from the basal end of the current-year shoot and then towards the apical end [2]. Meanwhile, on the contrary, there is bud retention in low crop load (“OFF”) years. Bud abscission is considered the visible mechanism underlining the alternate bearing [2].

**Figure 1:**
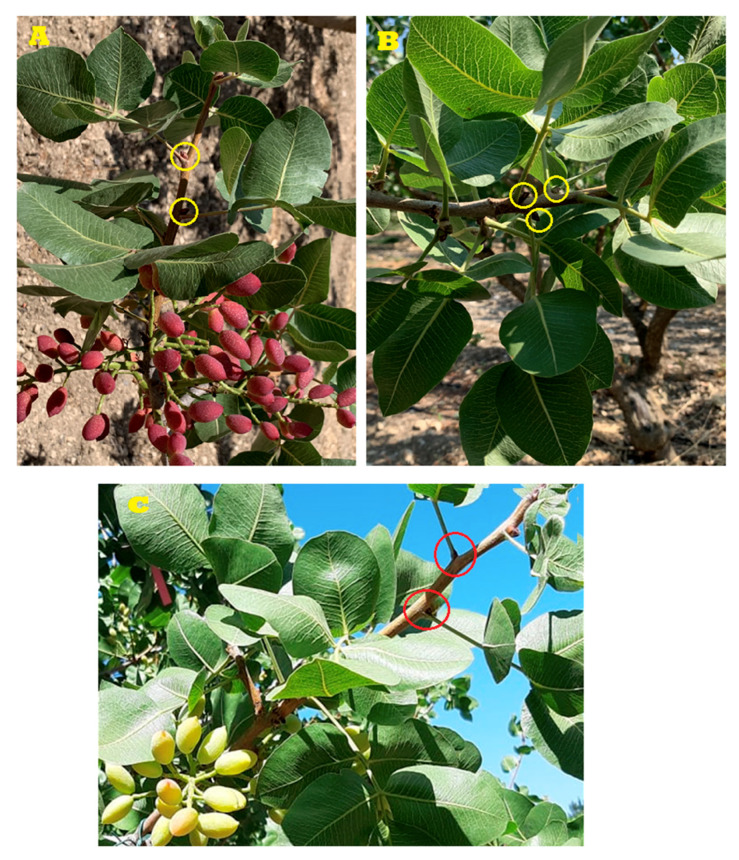
(**A**): Fruit clusters on one-year-old wood and lateral inflorescence buds on current year’s bearing shoot of *P. vera* L. indicated by yellow circles (June “ON”); (**B**): one-year-old wood and lateral inflorescence buds on current year’s not-bearing shoot (July “OFF”); (**C**): Red circles show the sites of inflorescence bud abscission in current year’s bearing shoot (July “ON”).

In order to simplify this phenomenon by outlining a time-line, lower buds start to abscise or drop at the end of June and continues in July and August, determining the heavy reduction in production in the next year, thus resulting in an “OFF” year. The figure below shows the growth pattern of the shoot during the “ON” and “OFF” season (Figure 1).

The physiological mechanism, which triggers the inflorescence buds drop linked to the alternate bearing behavior in pistachio, is not completely clear and two hypotheses are considered, one of which involves nutritional factors and the other involves hormonal factors. On the basis of the nutritional hypothesis, the competition of the growing embryos with the new inflorescence buds for the use of metabolites, carbohydrates and nitrogen can be the main cause of inflorescence bud dropping [5,6]. The hormonal hypothesis suggests that some growth regulators are directly involved in bud abscission. However, subsequent studies conducted on the levels of abscisic acid (ABA) in fruits and inflorescence buds did not show any relationship between the levels of this hormone and the bud drop [2,7].

The nutritional theory suggests that the inflorescence bud drop occurs in coincidence with the period of embryo growth and is more intense when the crop on one-year-old shoot is heavy, since the embryo represents the strongest “sink” [8]. This temporal coincidence of bud drops, and nut development suggests a competition between the developing embryo and inflorescence buds for the available resources. The lack of competitive ability of inflorescence buds compared to fruit in attracting the photosynthates produced by the leaves was demonstrated by tracking the translocation of the radioactive carbon isotope, C14 [7]. It also confirmed that in branches that are subjected to annular decortications, also known as shoot girdling (removal of a bark ring from the base of the current year’s shoot to separate it from the fruitescences), it is possible to reduce the inflorescence bud drop by 70% [9]. Similar results emerged from a study on the accumulation of nitrogen, phosphorus and potassium on the various organs of the branch, showing that the inflorescence buds of non-bearing branches accumulated significantly greater quantities of macro elements, compared to the inflorescence buds of the bearing branches [10].

Various studies have also highlighted the direct correlation between the fruit load and the intensity of the drop of the inflorescence buds [1,2,3,5,11]. Studies proved that the presence of the infructescence decrease the growth of the leaves and of the shoot axis [11,12], and that plants deprived of the fruits for the next years accumulate more carbohydrates and thus they express a greater potential for growth compared to those left in the normal year production cycle [13]. Many studies showed significant changes in starch content and the difference in translocation of starch in tissues of “ON” and “OFF” trees [2,8,9,11,12,14].

Moreover, in pistachio, within the canopies of “OFF” trees, it is possible to find some “ON” shoots and within canopies of “ON” trees, there always some “OFF” shoots; therefore, the theory of shoot or branch autonomy should be considered [2,7]. Shoot autonomy in fruit trees depends on resource (carbon, water, nutrients and hormone distribution) availability. These results underline the importance of reserve substances which, although stored in the permanent organs of the plant during the “OFF” year, are not enough for the full expression of the vegetative growth potential and the fructification. Interesting results have emerged from the study of the influence of polyamines (putrescine, spermine and spermidine) on the inflorescence bud drops [15]. In general, the level of polyamines is negatively correlated with inflorescence bud drops.

Genetic mechanisms involved in alternate bearing have been recently studied by transcriptomic analyses in some fruit crops, such as apple [16], citrus [17] and olive [18], where the alternate bearing is explained as the lack of flower bud initiation and their morphological differentiation, unlike in pistachio. In citrus, the fruit load critically affects bud fate before that flower induction occurs and an alternate bearing signal may be generated in the fruit or in another organ that perceives the flowering initiation and the change of key metabolic pathways [17]. It has been demonstrated in many fruit crops that “ON” and “OFF” crop status is associated with changes in the expression of flowering control genes [16,18]. Genes regulating trehalose and flavonoid metabolism and genes homologous to Squamosa promoter binding-like (SPL) were found induced in “OFF” buds of citrus [17].

In apple, microarray analysis showed that flower induction genes were differentially regulated between “ON” and “OFF” inflorescence buds and critical changes occur in expression of genes involved in oxidative stress, cell wall biogenesis, carbohydrate biosynthesis and lipid metabolism [16]. In olive, a cDNA library experiment performed on different developmental stages of leaves and fruits in “ON” and “OFF” trees showed that P450 monooxygenase and two dehydrins were more expressed in leaves of “ON” trees than in leaves of “OFF” trees [16]. Furthermore, in “ON” olive trees, a UDP-glucose epimerase, an acyl-CoA binding protein, a triose phosphate isomerase and a putative nuclear core anchor protein were more expressed in fruits. In “ON” and “OFF” olive trees, differences in miRNA-targeted genes were also found involved in main hormone signal-transduction pathways and carbohydrate metabolism which can be potentially associated in alternate bearing processes [18].

Preliminary transcriptional analysis in pistachio based in only a year of observation showed that in inflorescence buds of “ON” bearing shoots, photosynthesis related genes were down-regulated and some terpenoids related genes were up-regulated [19]. This study can be considered as the first study reporting and documenting the gene expression profiling associated with inflorescence bud abscission. Studies proved that genes associated with bud abscission may involve in alternate bearing directly or indirectly [2]. The aim of this analysis is to provide insights into the transcript changes between inflorescence buds in bearing and non-bearing shoots in order to identify the molecular mechanism causing premature inflorescence bud abscission, which is linked to alternate bearing in the Italian pistachio cultivar “Bianca”. Our results demonstrate the nutritional theory and the involvement of a complex network of hormonal signals and cross talk in the inflorescence bud drops of fruiting shoots. These findings have important implications for the horticultural management of this fruit species and can help in the breeding strategies for selecting the parents to cross the pistachio tree.

## 2. Materials and Methods

### 2.1. Plant Material, RNA Extraction, Processing and Sequencing

The transcriptomic analysis was conducted taking the tissue samples from one mature *P. vera* (L.) tree of the cultivar “Bianca”, grown inland of Sicily (37°30′ Lat. N), in 27th of June 2018 and in 22nd of July 2019. The inflorescence buds from bearing (“ON”) and non-bearing (“OFF”) branches were analyzed. Bearing branches showed from 40 to 50 fruits; non-bearing had no fruits. We collected 4–6 inflorescence buds each from three branches (considered as three biological replicates) of the same tree during the “ON” and “OFF” status of June and July which constitute a total of 12 samples. All bud samples were immediately frozen in liquid nitrogen after collection and stored at −80 °C. The samples were grounded in liquid nitrogen and total RNA extraction was performed with the Spectrum^TM^ Plant Total RNA Kit (Sigma-Aldrich, Milan, Italy) employing 100 mg of frozen tissue. RNA quality and RNA Integrity Number (RIN) were checked by using the Bioanalyzer. Libraries were obtained using the TruSeq RNA-Seq sample prep kit from Illumina (Illumina, Inc., San Diego, CA, USA). The 12 samples were loaded into one lane of an Illumina flow cell, and clusters were created by Illumina Bot. The clusters were sequenced using the service provided by BMR Genomics (Padua, Italy) at ultra-high throughput on the Illumina HiSeq 2000 (Illumina Inc.) to obtain single reads per sample, each 75 bp long.

### 2.2. De Novo Assembly, Evaluation and Annotation

The quality of the raw sequences generated from transcriptome sequencing was assessed with FastQC (version 1.16) (https://www.bioinformatics.babraham.ac.uk/projects/fastqc/). With respect to the FastQC report, the low-quality bases (Q-score < 30) were removed using custom made Perl script and the adaptor sequences were removed using cutadapt (version 2.0). The filtered reads were then aligned against Silva database (https://www.arb-silva.de/) using bowtie [20] (version 2.3.4.1) in order to remove rRNA reads and to obtain clean reads. The total pre-processed reads from all 12 samples were then de novo assembled using Trinity [21] (version 2.8.4) using default parameters. The transcripts from Trinity assembly were further clustered using CD-Hit-EST [22] (version 4.6.8), using a clustering threshold of 98% identity to reduce redundancy.

The assembly statistics were obtained using the transrate [23] (http://hibberdlab.com/transrate/) program. The assembly was evaluated with BUSCO [24] (version 3.0.2), a tool that assesses genome completeness based on the presence of single-copy orthologs, using the green plant dataset (viridiplantae_odb10). The complete assembly statistics and evaluation statistics is given in Table S1. The complete workflow of the Pistachio de novo transcriptome assembly and annotation were summarized in Figure S1.

The obtained contigs were annotated using BLASTx program (http://www.ncbi.nlm.nih.gov/BLAST/) with an E-value threshold of 1 × 10^−5^ to NCBI nr database (https://www.ncbi.nlm.nih.gov/refseq/about/nonredundantproteins), UniProt protein database (https://www.uniprot.org), InterPro database (https://www.ebi.ac.uk/interpro/), KEGG database (http://www.genome.jp/kegg), PFAM database (https://pfam.xfam.org) and STRING database (https://string-db.org). We considered only the contigs corresponds to ‘Viridiplantae’ and the unannotated contigs for the final transcriptome assembly. RNA-Seq data were deposited in NCBI’s sequence read archive (SRA) under accession number PRJNA623387.

### 2.3. Differentially Expressed Genes (DEG) between Stages

To estimate the expression levels of the Trinity reconstructed transcripts, we used RSEM [25]. RSEM is a package used to estimate the gene and isoform expression levels from RNA sequence data. The expected count matrix derived from RSEM is given as the input for edgeR [26]. The comparison selected for the study is given in Table 1. Genes represented with an adjusted *p*-value (FDR) lower than 0.01 and at least a two-fold change were only considered as significantly differentially expressed in the pairwise comparison of the samples. In addition, the functional-enrichment analysis was performed to identify which gene ontology (GO) terms and metabolic pathways that were significantly enriched in differentially expressed genes (DEGs).

### 2.4. Gene Enrichment and Functional Analysis

The final contigs were aligned against TAIR10 (https://www.arabidopsis.org) protein sequence using blastx program, in order to get the corresponding TAIR Id. The blastx result files were parsed and generated a Pistachio mapping file for Mapman containing the five categories (a) Nearly identical: Score ≥ 1000 and e-value = 0 (b) Highly similar: Score ≥ 1000 and e-value ≠ 0 OR (Score ≥ 500 & Score < 1000) and e-value = 0 (c) Moderately similar: (Score ≥ 200 & Score < 1000) and e-value ! = 0 (d) Weakly similar: (Score ≥ 100 & score < 200) (e) Very weakly similar: (Score < 100) based on the blastx score and e-value. The MapMan mapping file of Pistachio is available from https://drive.google.com/file/d/1nMp2euy36JwVtXIrVibEdnkaVB_AV2Uv/view.

We used MapMan (http://mapman.gabipd.org/) [27] with the Pistachio mapping file to map the gene IDs and visualize the metabolic overview, hormone regulation, CHO metabolism, secondary metabolism and transcription factors using two generated files: (1) related to “ON” and “OFF” stages of bud, (2) related to two time-point (June and July).

The PageMan analysis plugin of MapMan was used to visualize differences among metabolic pathways using Wilcoxon tests, no correction, and an over-representation analysis (ORA) cutoff value of 3. We considered all the differentially expressed genes present that are related to the comparison of “ON” and “OFF”, June and July for the PageMan analysis.

The TAIR IDs were searched against the Database for Annotation, Visualization and Integrated Discovery (DAVID) version 6.8 [28] Web server (https://david.ncifcrf.gov/). The gene ontology information related to the biological process was extracted from the DAVID result.

## 3. Results

### 3.1. De Novo Transcriptome Assembly and Annotation

High-throughput sequencing technology has provided an excellent opportunity for transcriptome survey in non-model plant species, including *P. vera.* To examine the inflorescence bud abscission phenomenon of *P. vera* relate to alternate bearing, inflorescence buds from three separate shoots of the same tree were collected and sequenced from bearing and non-bearing shoots. The picking dates of the material coincide with a period of the initial competition between fruit and inflorescence buds, not causing inflorescence bud drop, and a period of strong completion, causing the drop of inflorescence buds.

The sequencing of the data of June produced 199 million raw reads (60 Gb of data), whereas July produced 196 million reads (59 Gb of data) as a single-end. The high-quality single-end reads with an average quality score of 38 were selected for the transcriptome assembly after trimming off the low-quality bases and adapters from the June and July data sets (Table S2). The total pre-processed reads were then de novo assembled using Trinity and transcripts from Trinity assembly were further clustered using CD-Hit-EST. The assembly was evaluated with BUSCO to assess the transcriptome assembly by measuring the completeness of the transcriptome based on evolutionary present universal single-copy orthologs. The number of transcripts, mean length, N50 length, BUSCO scores and percentage of the alignment with the pre-processed reads were given in Table S1.

The number of up- and down-regulated genes, along with the total number of genes obtained in each sample comparison, are listed in Table 1.

We used RSEM for the quantification of the genes. The count matrix generated we then taken as the input by edgeR. The count matrix along with the expected count, FPKM and TPM are given in Table S3. The downstream analysis resulted in the identification of a total of 14,330 genes in which 4755 were up-regulated and 9575 were down-regulated. For each of the analysis, the total number of genes ranged from 1087 to 3882. The number of genes up-regulated was in a range of 247 to 1409 and down-regulated genes were spans from 435 to 3170. Subsequently, the assembled transcripts were annotated by BLASTX against a non-redundant (NR) protein database, PFAM, KEGG, Uniprot/Swissprot, InterPro and STRING databases. It is likely that the cv. Bianca faces a limitation of resources around the third week of June, when the first sampling of the plant material was made and, that in a month, it reaches its maximum peak, corresponding to the second sampling period when the drop of inflorescence buds started. June “OFF” vs. July “ON” corresponds to the most divergent scenarios. The Venn diagram shows the overlap of the genes for “ON” and “OFF” seasons of June and July (Figure S2). The figure shows 37,453 genes were common among all the seasons which might have a role in the developmental process rather than the alternate bearing. The main comparison is focused on “ON” and “OFF” period of June to investigate thoroughly different pathways and processes of the bud abscission. This comparison can also avoid factors like physiological and developmental changes that might occur in the bud during the two different time points (June and July). To add strength to our conclusion, a comparative study on the effect of crop load during the “OFF” and “ON” period of July is also investigated.

### 3.2. Effect of Crop Load on Photosynthesis During in June “OFF” vs. June “ON” Inflorescence Buds

The changes in gene expression in “ON” inflorescence buds do not reflect an enhancement of photosynthetic activity when compared to “OFF” year inflorescence bud from non-fruiting shoots). Most of the genes involved in photosynthesis were up-regulated during the “OFF” year. Both photosystem II PSII polypeptide subunits, MAF1, a global repressor of RNA polymerase III (Pol III) and PDE335 (Pigment defective 335) showed an up-regulation during June “OFF” (Table S4). In contrast, a gene calling for CRR3 (chloro-respiratory reduction 3) was repressed during the “OFF” period. The genes encoding for cytochrome (UGT76D1), ATP synthase (PDE332) and cyclic electron flow (PGR5-LIKE A) were enhanced during the June “OFF” period. (Table S4).

### 3.3. Effect of Crop Load on Starch Metabolism in—June “OFF” vs. June “ON” Inflorescence Buds

**Figure 2:**
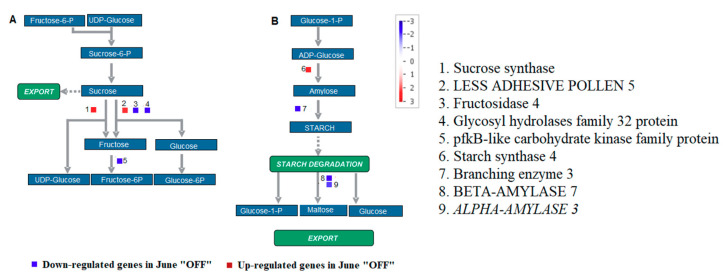
Figure shows the Mapman pathways in sucrose-starch metabolism. Figure highlights differentially expressed genes between inflorescence buds in the non-bearing shoot (June “OFF”) and inflorescence buds in bearing shoot (June “ON”) in sucrose degradation (**A**) and starch synthesis (**B**) pathways. Individual genes were represented by small squares. The color scale indicates the log2 FC value. Red represents up-regulation and blue represents down-regulation in June “OFF” relative to June “ON”.

Crop load had much greater effects in reducing starch contents and limiting the starch accumulation. Therefore, the study on the relationship between crop load and starch metabolism helps in assessing the functional distribution of starch in “ON” and “OFF” flower buds. The genes encoded for sucrose phosphate synthase (SPS1F), less adhesive pollen 5 and starch synthase 4 were enhanced in the tissue of inflorescence buds of the “OFF” current year shoots (Figure 2). These enzymes are involved in the formation of carbohydrate reserves [29]. The genes encoded *ALPHA-AMYLASE 3*, BETA-AMYLASE 7, fructosidase 4, glycosyl hydrolases family 32 and pfkB like carbohydrate kinase, which was involved in carbohydrate degradation was repressed (Figure 2).

### 3.4. Effect of Crop Load Status on Polyamine and Transcription Factors in Inflorescence Buds June “OFF” vs. June “ON” Shoots

This section of study was conducted to examine the role of free polyamines in the inflorescence bud abscission. The “OFF” inflorescence buds exhibited significantly higher polyamine (PA) and spermidine (Spd) enhancement than the “ON” ones, during most of the period (Figure S3). In “OFF” inflorescence buds, the genes encoding for thermospermine synthases (ACL5), probable polyamine transporter, Polyamine oxidase 1 isoform 1 and spermidine synthase (speE) were enhanced. On the contrary, the expression of S-adenosyl-L-methionine-dependent methyltransferases was repressed.

In June “OFF” inflorescence buds, three bZIP (bZIP61, leucine zipper transcription factor 16 and G-box binding factor 3), ARF7 (Auxin response factor 7), WRKY19, zinc ion binding and four homeobox genes (Enhanced drought tolerance 1, BEL1-like homeodomain 3, IFL1 and HB-8) were down-regulated, while one histone (ULI3) gene, two alfin-like members, two MYB factor (MYB103 and MYB14), three WRKY (WRKY31, WRKY72, and WRKY53), all the histone related factors and AS2 were up-regulated (Table S5). In June “ON” buds, the study reported the enhancement of MYB factors (MYB60, MYB3 and MYB106), WRKY factors (WRKY19 and WRKY49) and Histone acetyltransferases.

### 3.5. Gene Set and Pathway Enrichment Analysis During in June “OFF” vs. June “ON” Inflorescence Buds

DAVID software was used to identify the biological processes, cellular components and molecular functions affected by crop load at transcriptomic level considering the differentially expressed genes in the June “OFF” and June “ON”. While comparing, June “OFF” buds and June “ON” buds, 53 GO terms were down-regulated, whereas 31 were up-regulated (Figure S4). The biological pathways that are known to be repressed during “OFF” response to salicylic acid, chloroplast envelope, circadian rhythm, response to auxin, ion transmembrane transport, apoplast and proteolysis were found in our analysis. In contrast, we identified some GO-terms that were up-regulated in response to alternate bearing, such as nutrients ion transport, ABA catabolic process, gibberellin catabolic process, amino acid transmembrane transport and carbohydrate metabolic process (Figure S4).

### 3.6. Effect of Crop Load on Hormone Metabolism in June “OFF” vs. June “ON” Inflorescence Buds

**Figure 3:**
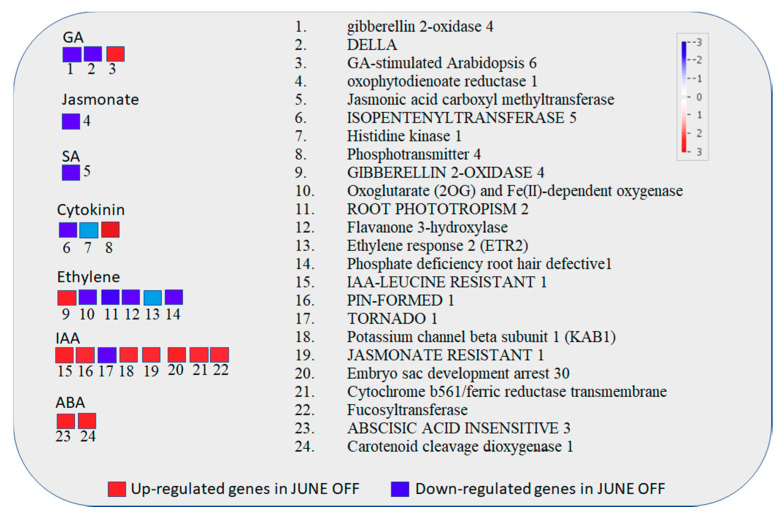
Figure shows hormone metabolism in Pistachio among the June “OFF” vs. June “ON” inflorescence bud comparison. The color scale indicates the log2 FC value. Red represents up-regulation and blue represents down-regulation in June “OFF” buds relative to June “ON” buds.

The objective of the current section was to study the role of hormone in inflorescence bud abscission. The genes involved in hormone-related categories are summarized in Figure 3.

Repression of ethylene and gibberellin pathways were identified in inflorescence buds of “OFF”, whereas ABA and IAA pathways were mostly up-regulated. In June “OFF” inflorescence buds, five genes responsive to ethylene, two genes responsive to gibberellin and two genes responsive to cytokinin were down-regulated (Figure 3). Relating to auxin-responsive genes, down-regulation of PIN formed 1 and up-regulation of TOR, Potassium channel beta subunit 1 (KAB1) and Jasmonate resistant 1 were observed. Relating to ABA there was an up-regulation in abscisic acid insensitive 3, lipid transfer protein 3, shaker potassium ion channel, SNF1, potassium transport 3, phosphotransmitter 4 and Carotenoid cleavage dioxygenase 1. Several genes involved in ethylene biosynthesis and signaling such as Oxoglutarate (2OG) and Fe (II)-dependent oxygenase, Root phototropism 2, Flavanone 3-hydroxylase, Ethylene response 2 and phosphate deficiency root hair defective 1 were repressed during in “OFF” buds (Figure 3).

### 3.7. Effect of Crop Load on Ubiquitin and Autophagy Dependent Degradation in June “OFF” vs. June “ON” Inflorescence Buds

**Figure 4:**
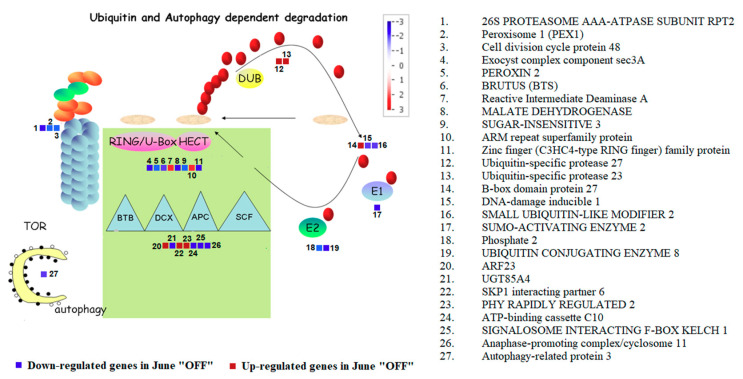
Figure shows Ubiquitin and Autophagy dependent degradation in Pistachio among the June “OFF” vs. June “ON” inflorescence bud comparison. The color scale indicates the log2 FC value. Red squares represent up-regulation and blue squares represent down-regulation in June “OFF” relative to June “ON”.

The results on the effect of crop load on ubiquitin and autophagy-dependent degradation could be a tool for understanding the premature inflorescence bud abscission presumably associated to the alternate bearing mechanism of *P. vera*. The genes in inflorescence buds from “ON” and “OFF” shoots, that were involved in ubiquitin and autophagy-dependent categories were summarized in Figure 4.

It is worthy to mention that most of the genes were repressed in “OFF” inflorescence buds. During this season, genes responsive to autophagy (ATG8C: Autophagy related protein 3) and genes responsive to ubiquitin proteasome (PAG1: Proteasome Alpha Subunit G1 and Cytochrome P450) were down-regulated. While discussing the E3 RING/U-BOX genes, it is worthy to mention the down-regulationof PEROXIN 2, BRUTUS (BTS), Malate dehydrogenase, Sugar-insensitive 3 and MAPK in OFF buds. Relating to the up-regulated genes, we noticed genes such as reactive intermediate deaminase A, ARM repeat superfamily protein, B-box domain protein 27, ARF23, SKP1 interacting partner 6 and PHY rapidly regulated 2 (Figure 4).

### 3.8. Effect of Crop Load on Carbohydrate Metabolism and Mobilization in June “OFF” vs. June “ON” Inflorescence Buds

**Figure 5:**
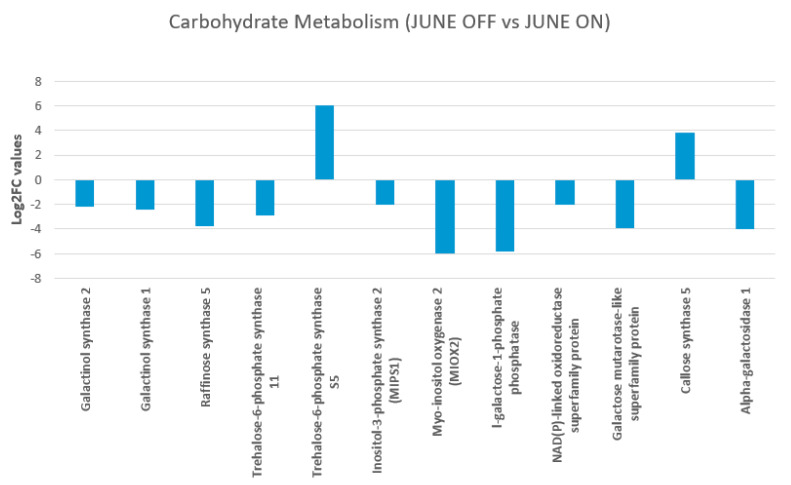
Figure shows carbohydrate metabolism and mobilization pathway in Pistachio among the June “OFF” vs June “ON” inflorescence bud comparison. The red circle represents the value of log2 fold change. The line indicates the effects on carbohydrate levels driven by differential expression of different CHO metabolism genes.

The objective of the current section was to verify the role of CHO reserves and mobilization as a cause or effect of the drop of inflorescence buds in *P. vera*. The relationship among the carbohydrate metabolism and mobilization pathway in Pistachio and the inflorescence buds from non-bearing shoots (June “OFF”) and bearing shoots, June “ON” is indicated in Figure 5.

The study showed that the pistachio inflorescence bud of non-fruiting shoots “OFF” required low amounts of carbohydrates due to the lack of fruits at the time and thus accumulated some starch. Similarly, we also found that the inflorescence buds of bearing and non-bearing pistachio shoots differed in their carbohydrate storage and mobilization patterns, suggesting that the in-season carbon mobilization might influence the flower bud abscission directly or indirectly linked to the alternate bearing. Raffinose synthase gene (Raffinose synthase 5 (RS5), two galactinol synthase genes (Galactinol synthase 1 and Galactinol synthase 2) and MIOX2 showed repression in the “OFF” buds, whereas sugar alcohols, such as callose synthase and trehalose-6-phosphate synthase 11, showed an up-regulation (Figure 5).

### 3.9. Comparison between “ON” and “OFF” Inflorescence Buds of “JUNE” and “JULY”

A comparative study of differently regulated genes among the “ON” and “OFF” inflorescence buds collected in “June” and “July” summarizes that, during the “OFF” season of July, there is a gradual reduction of raffinose synthase 1 and MIOX2 as we identified in June “OFF”. The enhancement of hormones like ABA and, at the same time, reduction of gibberellin and ethylene indicates that July “OFF” is gradually showing the same pattern as that of June “OFF” (Figure S5). SnRK1 and TOR down-regulated both the cases; therefore, no programmed cell death (PCD) and autophagy occurs during July “OFF” and makes plant stable for the next upcoming “ON” season. During the comparison of the inflorescence buds in fruiting shoots “ON” of June and July, we could find that almost all the genes during July “ON” participate in a similar way as that of “ON” June. This comparison proves that gene expression profiling associated with “ON” season of June and July and “OFF” season of June and July are similar proving the importance of these genes in the flower bud abscission and alternate bearing (Figure S5).

#### Effects of Crop Load in July “OFF” vs. July “ON” Inflorescence Buds

An enhancement ABA was identified in inflorescence buds of July “OFF”, whereas ethylene and gibberellin pathways were mostly down-regulated. In July “OFF” inflorescence buds, three genes responsive to ethylene (ETR2, 2-oxoglutarate (2OG) and Fe(II)-dependent oxygenase, Flavanone 3-hydroxylase) one gene responsive to gibberellin (gibberellin 2-oxidase 4) and one gene responsive to cytokinin (Isopentenyl transferase 5) were down-regulated (Figure S6). Relating to auxin-responsive genes, down-regulation of PIN formed 1 and auxin F-box protein 5 and the up-regulation of TOR, Potassium channel beta subunit 1 (KAB1) and Glycoside Hydrolase Family 3 were observed. Relating to ABA there was an up-regulation in SNF1, Abscisic acid insensitive 3 and Carotenoid cleavage dioxygenase 1 (Figure S6).

The comparative study of the July “OFF” vs July “ON” produced similar results to the results of June “OFF” vs June “ON”. The genes encoded for sucrose phosphate synthase (SPS1F) and starch synthase 4 were enhanced in the inflorescence buds of the July “OFF”. The genes encoded *ALPHA-AMYLASE 3*, BETA-AMYLASE 7 and fructosidase 4 were repressed (Figure S7).

Most of the genes involved in photosynthesis were up-regulated during the July “OFF” year, similar to the results of June “OFF”. The photosystem II PSII polypeptide subunits and PDE335 (Pigment defective 335) showed an up-regulation, whereas gene calling for CRR3 (chloro-respiratory reduction 3) was repressed during July “OFF” (Table S6). The genes encoding for cytochrome (UGT76D1), ATP synthase (PDE332) and cyclic electron flow (PGR5-LIKE A) were enhanced during the “OFF” period (Table S6).

## 4. Discussion

The growth of the endocarp of the cultivar Bianca is from the first week of May to the end of June, while the growth of the embryo is from the first week of July to the end of August [1]. In “ON” trees, most of the inflorescence bud’s abscission starts at the end of June and continues in July and August. None of the works to date could confirm the involvement of flowering promoter and repressor genes in regulating inflorescence bud’s abscission in pistachio. This study provides insights into the transcript changes between inflorescence buds in bearing and non-bearing shoots in order to identify the molecular mechanism causing premature inflorescence bud abscission, which is linked to the alternate bearing in the Italian pistachio cultivar “Bianca”.

The relationships between the flower bud drops linked to alternate bearing and the carbohydrate storage have been mentioned in several studies [2,5,13,30]. It generally seems evident that in pistachio trees, nutrients are stored during the “OFF” year and that they are used for reproductive growth in the following year [2,13,14,31]. There are significant changes in starch content and different translocation of starch in the tissues of “ON” and “OFF” trees [14,32] and it has been suggested that the mobilization of stored carbohydrates may cause inflorescence bud abscission in pistachio [8]. The role of individual sugars in the process of inflorescence bud abscission has not yet been investigated [2].

In our study, genes encoding for sucrose phosphate synthase (SPS1F), degradation sucrose invertase (A/N-InvE), starch synthase 4, callose synthase and trehalose-6-phosphate synthase 11 were enhanced in the June “OFF” inflorescence buds, whereas BETA-AMYLASE 7, *ALPHA-AMYLASE 3*, branching enzyme 3, fructosidase 4, glycosyl hydrolases family 32, and pfkB like carbohydrate kinase were repressed. This supports the nutritional theory demonstrating that nutrients are stored during the “OFF” year to be used for reproductive growth the following year in pistachio trees. Interestingly, in rice and sugar, hormone signals regulated *ALPHA-AMYLASE 3* enzyme expression, which catalyzed starch degradation [33]. In particular, sugar starvation promoted the expression of *ALPHA-AMYLASE 3* that resulted in the up-regulation in pistachio “ON” inflorescence buds.

Raffinose synthase gene (Raffinose synthase 5 (RS5), two galactinol synthase genes (Galactinol synthase 1 and Galactinol synthase 2) and MIOX2 showed enhancement in our study in inflorescence buds of bearing shoots (“ON”). The raffinose family of oligosaccharides has a wide range of predicted functions and are currently emerging as crucial molecules during stress response in plants [34], because of their membrane-stabilizing, antioxidant and, perhaps, predictable signaling functions [35]. They participate in several cellular functions, such as transport and storage of sugars [36], signaling molecule following pathogen attack and wounding [37], signal transduction [38], membrane trafficking [39] and mRNA export [40]. Recent transcriptional profiling data in *Arabidopsis thaliana* showed up-regulation of the Myo-inositol oxygenase (MIOX) genes under limited energy or nutrient conditions shows consistent with our results indicating an up-regulation in “ON” buds. MIOX2 plays a prominent role in the oxidation of inositol for the needs of the plant in different tissues and it is involved in the biosynthesis of nucleotide sugar precursors for cell-wall matrix polysaccharides [41].

Interestingly, trehalose-6-phosphate (T6P), that was up-regulated in “OFF” inflorescence buds, seem to play a central role in sugar metabolism regulation in plants [42]. It has been proposed that T6P is transported by an unknown mechanism into plastids, where it induces starch synthesis via thioredoxin-mediated activation of AGPase, and that there is a regulatory loop which involves T6P, SnRK1 (a gene that represses plant growth, inhibited by T6P) and bZIP11 that control sucrose availability and utilization. In source leaves, T6P fine-tunes sucrose levels by adjusting sucrose synthesis, while it regulates Sucrose consumption in sink organs, probably acting via multiple mechanisms, including inhibition of the SnRK1 gene [43]. T6P regulates growth in relation to sucrose supply by adjusting biosynthetic reactions and through regulating hormone signaling like auxin either directly or indirectly [44].

In the pistachio tree, it has been demonstrated that the accumulation of nitrogen, phosphorus and potassium is greater in inflorescence buds of non-bearing branches, compared to the ones of the bearing branches [10], and that the nutrient contents of the trees and annual nutrient consumption are influenced by the alternate bearing [10]. Our results showed an enhancement of nitrogen permease regulator of amino acid transport activity 3 and carbon-nitrogen hydrolase, which is supporting the fact that the concentration of nitrogen (N) was higher in the inflorescence buds, leaves, and fruits of non-fruiting branches (OFF) than in the analogous “ON” structures [10]. Competition between flower buds and developing nuts for N might play an important role.

Our study showed up-regulation of potassium ion channel, magnesium dechelatase (SGR), magnesium-chelatase subunit (ChlH), CSC1-like protein (Calcium-dependent channel) and calcium permeable stress-gated cation channel (TMEM63) during in “OFF” inflorescence buds. Some studies found that N, K, Ca and Mg content were affected by crop load in olive leaves, showing lower values following the “ON” year [45]. However, the information on the effects of fruiting on nutrient concentrations of different organs of pistachio trees relative to bud abscission is limited.

In the “OFF” inflorescence buds of June, we found genes encoding for sugar phosphates accumulation including substrates of the Calvin cycle, glycolysis, and the pentose phosphate pathway. Sugar phosphates transformed into sucrose and transport to fruit. This can reduce the sugar phosphates in the source tissues of trees with strong sink tissue such as fruit. Whereas, in the inflorescence buds of “OFF” shoots, the absences of fruits lead to the accumulation of sugar phosphates and starch. Studies showed that the expression of some of the genes and proteins involved in the Calvin cycle is up-regulated in “OFF” trees [18].

Studies on the stomatal transpiration rates in another alternating species, such as the olive tree, have not shown any variation between plants in “ON” and “OFF” [46], contrary to what occurs in species like orange or strawberry [47]. Studies on photosynthesis and production of photosassimilate in pistachio have shown a decline in “ON” trees during mid-July, which could be due to early senescence and the fall of the leaves [48]. A similar decline in photosynthesis due to leaf aging has been reported for apple trees [49] and olive trees [46]. In the present study, the up-regulation of both the PSII polypeptide subunits of the photosystem II, MAF1, a global RNA polymerase III (Pol III) and PDE335 (defective Pigment 335) repressor that can be found in the “OFF” buds of June indicate that pistachios have the ability to maintain relatively high photosynthetic rates.

In plants, nutrient limitation due to sink competition leading to sugar starvation is perceived as nutritional stress and generate changes in the redox status promote the synthesis of free radicals which can cause transient oxidative stress due to an increase of ROS generation [50], that can be neutralized by some adaptive mechanisms which can protect the cells from oxidative damage. The cells subjected to sugar starvation at the beginning try to adapt to this deficiency through a gradual metabolic reorganization that implies the substitution of carbohydrate metabolism by protein and lipid metabolism and that change may cause autophagy [51]. Variations in sugar levels induce changes in ROS production and sugar starvation can cause the activation of ROS production, as indicated by transcriptome profiling analysis, where sucrose starvation results in activation of oxidative stress genes, such as catalase [52].

It has been found that plant processes, such as cell division, morphogenesis and stress responses, were affected by the involvement of polyamines (PAs)-putrescine (Put), spermidine (Spd), spermine (Spm), cadaverine (Cad) and thermospermine (t-Spm) [53]. The free polyamines could have an important physiological function in the development of flower bud abscission, which causes alternate bearing in pistachio trees [13]. A significant decrease in polyamines (Pas; Put and Spd) in shoots and leaves of “ON” trees during the heavy bud, abscission period was reported while an increase detected during the same period in “OFF” trees, indicating an association between flower bud abscission and the level of PAs in pistachio. In Satsuma mandarin, during the “ON” season, polyamines were accumulated in the stem which can suppress flowering and cause fruit bearing [54]. It is possible that a decrease in N concentrations in plant tissues may cause a decrease in polyamines, as they can represent nitrogenous sources or as signal molecules that regulate the fruitlet abscission process in grapevine [55]. Many studies have highlighted that abscission or ethylene biosynthesis can be delayed with low levels of S-adenosylmethionine (SAM). During this phase, PAs and ethylene compete and PAs can become dominant. The low concentrations of PAs can trigger the senescence and cause abscission [56].

In our study, polyamines related genes exhibited significantly higher enhancement in the inflorescence buds of non-fruiting branches (OFF) than the “ON” fruiting ones, in accord with the recent study of Gündeşlí [15]. The genes encoding for thermospermine synthases (ACL5), probable polyamine transporter, Polyamine oxidase 1 isoform 1 and spermidine synthase (speE) were enhanced during the “OFF” seasons. These references, along with our results, support the fact that polyamines could play a crucial role in the inflorescence bud abscission of pistachio. A high level of polyamines is known to act as antisenescence agents and counteract the activity of abscisic acid and ethylene [57]. The competition between polyamines and ethylene pathways for S-adenosil methionine (AdoMet) or the inhibition of ACC syntase or ethylene forming enzyme (EFE) by polyamines can result in a mechanism that can modulate physiological events, including senescence and flower bud abscission.

The role of hormonal factors involved in inflorescence bud abscission was studied by many authors in pistachio leading to contrasting results [58]; however, only recently lower levels of auxin in most of the organs of “ON” pistachio trees during kernel development have been directly implicated in bud abscission [59]. Exogenous application of auxins prevented inflorescence bud abscission in pistachio [60]. In another study conducted in citrus the auxin amount is in a positive relationship with abscission by causing a delay of abscission, resulting in improvement in fruit quality and yield. In our research, auxin was down-regulated in “ON” buds. Our study shows that auxin conjugates play an important role in IAA metabolism, temporary storage reserves and inflorescence bud abscission.

In the auxin-responsive gene category, differentially expressed in the present study, it is worth to mention the down regulation of TOR that we found in June “OFF” inflorescence buds and up-regulated in June “ON”. The regulation of autophagy by TOR and SnRK1 or SNF1-related kinase is conserved in plants [61]. In Arabidopsis, AuTophaGy-related1 (ATG1) kinase complex and ATG13 together generate a complex which can regulates autophagy, nitrogen deprivation and short-term carbon starvation. Furthermore, this ATG1-ATG13 complex are sensitive to the nutrient level mediated by TOR [62]. https://dev.biologists.org/content/145/13/dev160887—ref-115 SnRK1 complex is activated by energy deprivation, abiotic stresses and starvation but suppressed by glucose in Arabidopsis [63]. SnRK1 and TOR can target phosphorylation substrates to sense energy and nutrient levels and coordinate transcriptome, metabolism, cell growth and development [64]. Interestingly we found the down regulation of SnRK1 or SNF1-related kinase in June “OFF” inflorescence buds and an enhancement in June “ON”. TOR signaling plays an important role in stem and progenitor cell function and regulation that modulate proliferation and maintenance, cell-cell interactions and sink-source organ communication.

There are several studies reporting the involvement of ABA biosynthesis or ethylene perception critical for sugar signaling [65]. The ethylene signal is transmitted via a pathway that includes a transcriptional cascade, and EIN3 has been identified as a critical component within this cascade [66]. The regulation of EIN3 by ethylene and sugar indicates the cross talk between the two signaling pathways. Remarkably, we have found that the transcription of ethylene is also down-regulated by glucose in June “OFF” inflorescence buds, whereas ABA encoding genes like carotenoid cleavage dioxygenase 1 and abscisic acid insensitive 3 were up-regulated during June “OFF” inflorescence buds.

Interestingly, in barley, the antagonism between ABA and GA has been demonstrated to be an essential factor controlling the metabolism in aleurone cells and the PCD. GA induces the production of hydrogen peroxide (H_2_O_2_) and α-amylases in aleurone cells which lead to hydrolyse stored starch [67]. Thus, the high level of GA expression that we found in June “ON” inflorescence buds can be an indication of the shortage of sugar and a signal for inducing starch degradation to supply the carbohydrate need.

In studies on abiotic stress, responses showed the involvement of polyamines in PCD through the production of hydrogen peroxide (H_2_O_2_) and Nitrogen oxide (NO) [68]. Abiotic stress conditions induce an excess of spermidine into the apoplast, where it is catabolized by the enzyme PA oxidase, producing reactive oxygen species (ROS) such as H_2_O_2_ and/or other nitrogenous molecules (N) through different cascades [69]. H_2_O_2_ accumulation can cause the induction of PCD or stress tolerance, depending on the levels of intracellular Pas [70]. PCD is strictly regulated by the ratio of PA anabolism to catabolism, while ROS generation/accumulation has a crucial role in cell fate decision [71].

PA catabolism by amine oxidases, copper-containing amine oxidases (CuAOs), flavin-containing PA oxidases (PAOs) and the parallel production of H_2_O_2_ can result in two different scenarios. High H_2_O_2_ levels lead to programmed cell death (PCD) [72], while low H_2_O_2_ level is efficiently scavenged by enzymatic/nonenzymatic antioxidant factors that help plants to survive abiotic stress, using different defense mechanisms [73]. In the present study, ROS related genes such as peroxisome 1 (PEX1), isocitrate dehydrogenase, COPPER/ZINC SUPEROXIDE DISMUTASE, FLAVANONE 3-HYDROXYLASE, Peroxidase, HXXXD-type acyl-transferase family protein as well as many stress-related genes (Disease resistance protein, Glycosyl hydrolase, Cysteine-rich secretory proteins, Serine/threonine-protein kinase MAPK/ERK KINASE 4, VASCULAR ASSOCIATED DEATH1, Leucine-rich repeat protein kinase, Riboflavin synthase-like superfamily protein and Pentatricopeptide repeat) were found down-regulated in June “OFF” inflorescence buds vs June “ON” inflorescence buds. It is well known in both animals and plants that peroxisome *PEX* genes are induced by the universal stress signal, H_2_O_2_ [74].

Most of the genes involved in ubiquitin and autophagy dependent categories were repressed during the June “OFF” inflorescence buds, (ATG8C: Autophagy related protein 3, ubiquitin proteasome, PAG1: Proteasome Alpha Subunit G1, Cytochrome P450, E3 RING/U-BOX genes, PEROXIN 2, BRUTUS (BTS), Malate dehydrogenase, Sugar-insensitive 3 and MAPK genes). It has been found that BTS may act as an E3 ligase, which catalyzes the final step in the protein ubiquitination via the 26S proteasome [75]. During June “OFF” inflorescence buds, we noticed the up-regulation of reactive intermediate deaminase A, ARM repeat superfamily protein members of the U-Box E3 Ubiquitin Ligase Family, B-box domain protein 27, ARF23, SKP1 interacting partner 6 and PHY rapidly regulated 2.

In our study, three bZIP transcription factors were found to be down-regulated during the June “OFF” inflorescence buds. bZIP61 harbor various stress-related cis-elements, indicating this bZIP related gene may involve in response to multiple abiotic stresses. In rice, OsbZIP genes, like OsbZIP16, act as positive regulators of drought and osmotic stress [76]. The bZIP61 and bZIP16 were found up-regulated in June “ON” bud. The Auxin response factors were found to be down-regulated during the June “OFF” buds, while ARF7 was found up-regulated in June “ON” bud. In *A. thaliana* two related auxin response factors, ARF7 and ARF19 act as transcriptional activators of early auxin response genes during lateral root formation. Three WRKY (WRKY31, WRKY72 and WRKY53) transcription factors having a key role in response to many different environmental stresses were up-regulated in June “OFF” inflorescence buds. *AP2/EREBP* (APETALA2/ethylene-responsive element-binding protein) transcription factors were found up-regulated in June “ON” inflorescence buds. Interestingly, in rice, *OsAP2/EREBP* plays an important role in the crosstalk of signaling pathways of different kinds of stresses [77].

**Figure 6:**
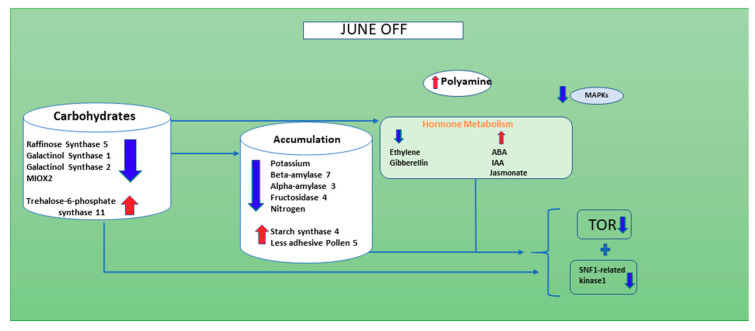
A figure showing the down-regulated genes in the inflorescence buds of non-fruiting branches of June “OFF” season. Red shows the up-regulated genes and blue shows the down-regulated genes. In this situation the inflorescence buds do not occur.

The evidence of transcriptomics results allowed the elaboration of a model that supports the nutritional theory and elucidates for the first time the role of hormones, polyamines and ROS in inflorescence buds abscission likely associated to the alternate bearing behavior of the pistachio. We can speculate that when the level of sugar is not critical, as indicated by the down-regulation of genes involved in starch demolition (*ALPHA-AMYLASE 3* and *ALPHA-AMYLASE 7*) and up-regulation of starch synthase 4, like in June “OFF” inflorescence buds, SnRK1 complex is suppressed by sugars or by trehalose-6-phosphate (T6P), considered a fine-tunes of sucrose levels [40], which is up-regulated and the transcription of ethylene and GA are down-regulated, as well as many stress relate genes and ubiquitin and autophagy-dependent genes. Auxin related genes, on the contrary, are up-regulated, indicating a possible accumulation of this hormone, inducing cell growth and perhaps the down-regulation of TOR. The oxidization of polyamines, such as Spd, occurs in the apoplast at a slow rate, with moderate production of H_2_O_2_, which activates the ROS-dependent protective pathway that does not trigger PCD or autophagy [78] (Figure 6).

**Figure 7:**
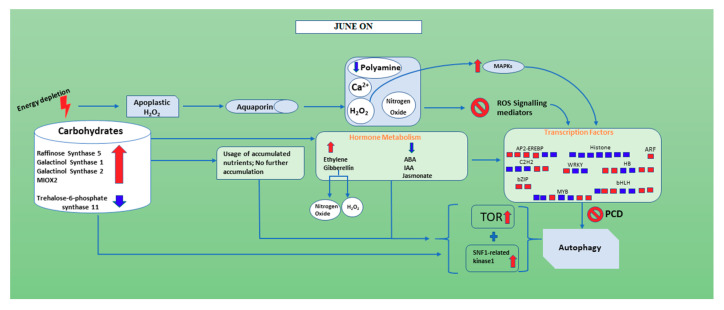
A figure showing the hypothetical molecular mechanism behind inflorescence buds abscission in fruiting branches of June “ON” season. Red and blue show the up-regulated and down-regulated genes in inflorescence buds of the June “ON” season, respectively.

In June “ON” inflorescence buds when the degradation of starch occurs as indicated by the up-regulation of *ALPHA-AMYLASE 3*, T6P is down-regulated and SNF1-related kinase 1 and TOR are activated. TOR signaling networks seem involved in cell-cell interactions, sink-source organ communication and autophagy [78]. In our study, Raffinose synthase gene 5 (RS5), galactinol synthase genes (Galactinol synthase 1 and Galactinol synthase 2) and MIOX2 showed enhancement in “ON” inflorescence buds. In June “ON” inflorescence buds, spermidine oxidation occurs faster with the high production of H_2_O_2_ inducing PCD pathway [71] and PA are down-regulated. Interestingly, genes of the GA pathway, up-regulated in June “ON”, may also increase the production of hydrogen peroxide (H_2_O_2_), which can represent a signal for inducing starch degradation to supply the carbohydrate need. Furthermore, in conjunction with low PA expression, down-regulation of auxin was also found resulting altogether in flower bud abscission (Figure 7). It is very interesting to note the enhancement of some transcription factors in July “ON”, which presumably increase programmed cell death and autophagy by promoting a more substantial abscission of inflorescence buds since the depletion of nutrients is greater due to the intense growth of embryos (Figure S5).

This work concludes that, in the “OFF” inflorescence buds of June, the genes corresponding to carbohydrate show reduction compared to June “ON” inflorescence bud. Furthermore, there is a higher amount of accumulation of starch (BETA-AMYLASE 7, *ALPHA-AMYLASE 3* and Fructosidase 4), nitrogen and potassium in June “OFF” compared to June “ON” (Figure 6). The hormones such as ethylene and gibberellin are showing down-regulation and ABA, IAA and Jasmonate are showing up-regulation when compared with June “ON” inflorescence buds; we can conclude that these hormones play an important in the production of Nitrogen oxide, Polyamine and H_2_O_2_, which eventually target cell death and autophagy during the June “ON” period. As predicted, there is no such signaling taking place for H_2_O_2_ and ROS and polyamines show change towards its enhancement in June “OFF”. Therefore, during June “OFF” inflorescence buds, no PCD and autophagy occur (Figure 6) and makes plant stable for the next upcoming “ON” season (Figure 7). Interestingly it seems that in pistachio exogenous application of PA can reduce many physiological disorders and inflorescence bud abscission [57,79], and preliminary experiments are currently being carried out in the cultivar Bianca to detect the dose and the timing of treatment.

These results highlighted how the lack of resources (carbohydrates and mineral elements) in *P. vera* can be the main cause triggering a cascade of events involving hormones and ROS which end, through autophagy phenomena, with the abscission of inflorescence buds, directly or indirectly linked to the mechanism of alternating production. This study provided further support to the theory of shoot autonomy in pistachio with regards to flower bud abscission and identified key genes and hormones associated with inflorescence bud abscission, the knowledge of which could also lead, in future, to a reduction of the inflorescence buds drop, through the development of biomarkers, and the possibility to modulate the alternate bearing.

## Figures and Tables

**Table 1:** The number of total genes, up-regulated and down-regulated genes in inflorescence buds in current year non-fruiting shoot “OFF” and in inflorescence buds in fruiting shoots “ON”.

Comparison (Inflorescence Bud)	Differentially Expressed Genes	Up-Regulated	Down-Regulated
July “OFF” vs. July “ON”	1087	247	840
June “OFF” vs. July “OFF”	2299	976	1323
June “ON” vs. July “OFF”	2450	591	1859
June “OFF” vs. July “ON”	2768	820	1948
June “ON” vs. July “ON”	3882	712	3170
June “OFF” vs. June “ON”	1844	1409	435

## Supplementary Materials

The following are available online at https://www.mdpi.com/2073-4425/11/8/851/s1. Table S1: The table shows the de novo assembly information. Table S2: The details of the sequencing and adapter trimmed data. Table S3: The table shows differentially expressed genes encoding for photosynthetic pathway in pistachio inflorescence buds of non-fruiting shoot June “OFF” vs. fruiting shoot June “ON”. Table S4: The output generated from RSEM which includes gene id, expected counts, FPKM and TPM. Table S5: The table shows differentially expressed genes encoding for transcription factors in pistachio inflorescence buds among the June “OFF” vs. June “ON” comparison. Table S6: The table shows differentially expressed genes encoding for photosynthetic pathway in pistachio inflorescence buds of non-fruiting shoot July “OFF” vs. fruiting shoot July “ON”. Figure S1: Workflow of the de-novo analysis of the transcriptomic studies related with inflorescence bud abscission in bud tissue. Figure S2: The Venn-diagram shows the overlap of the genes for “ON” and “OFF” inflorescence buds of June and July. Figure S3: Figure shows polyamine biosynthesis pathway in pistachio inflorescence buds among the June “OFF” vs. June “ON” comparison. The color scale indicates the log2 FC value. Red represents up-regulation and blue represents down-regulation in June “OFF” relative to June “ON” buds. Arrows indicate the effects on PA levels driven by overexpression of different PA biosynthesis genes. Figure S4: The bar plot showing gene set and pathway enrichment analysis in pistachio buds among the June “OFF” vs. June “ON” inflorescence buds’ comparison. Figure S5: The figure showing the hypothetical molecular mechanism behind inflorescence buds abscission in fruiting branches of July “ON” and “OFF” season. Red and blue show the up-regulated and down-regulated genes in inflorescence buds. Figure S6: Figure shows hormone metabolism in Pistachio among the July “OFF” vs. July “ON” inflorescence bud comparison. The color scale indicates the log2 FC value. Red represents up-regulation and blue represents down-regulation in July “OFF” inflorescence buds relative to July “ON” inflorescence buds. Figure S7: Figure highlights differentially expressed genes between inflorescence buds in non-bearing shoot (July “OFF”) and inflorescence buds in bearing shoot (July “ON”) in sucrose degradation (A) and starch synthesis (B) pathways. Individual genes were represented by small squares. The color scale indicates the log2 FC value. Red represents up-regulation and blue represents down-regulation in July “OFF” relative to July “ON”.
