# Supplementary figures and images for: Transcriptome Analysis of Pistacia vera Inflorescence Buds in Bearing and Non-Bearing Shoots Reveals the Molecular Mechanism Causing Premature Flower Bud Abscission
Source: Genes (Basel). 2020 Jul 25;11(8):851. doi: 10.3390/genes11080851 (PMC7465039; doi:10.3390/genes11080851)

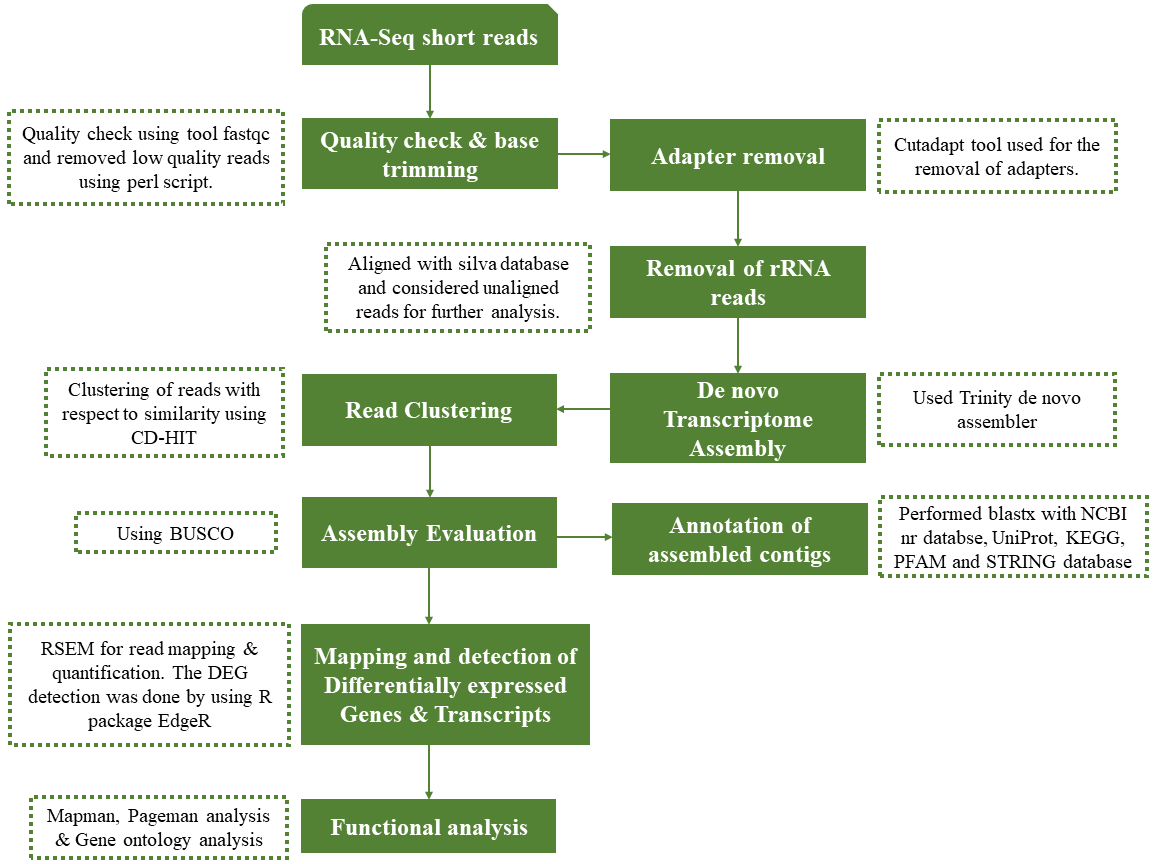

Supplement: Supplementary file 1 [file genes-11-00851-s001.zip › Supplementary_Files/Figure S1.tif]

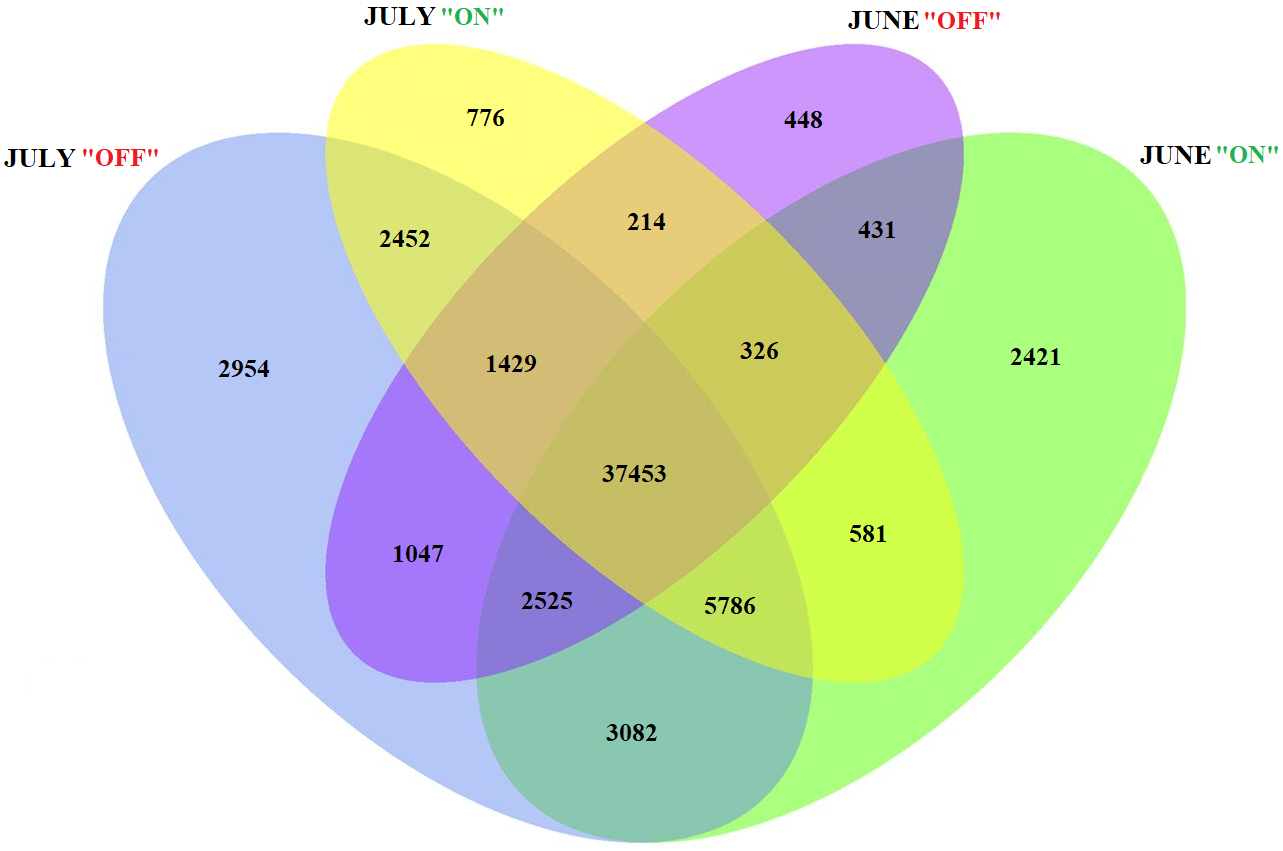

Supplement: Supplementary file 1 [file genes-11-00851-s001.zip › Supplementary_Files/Figure S2.tif]

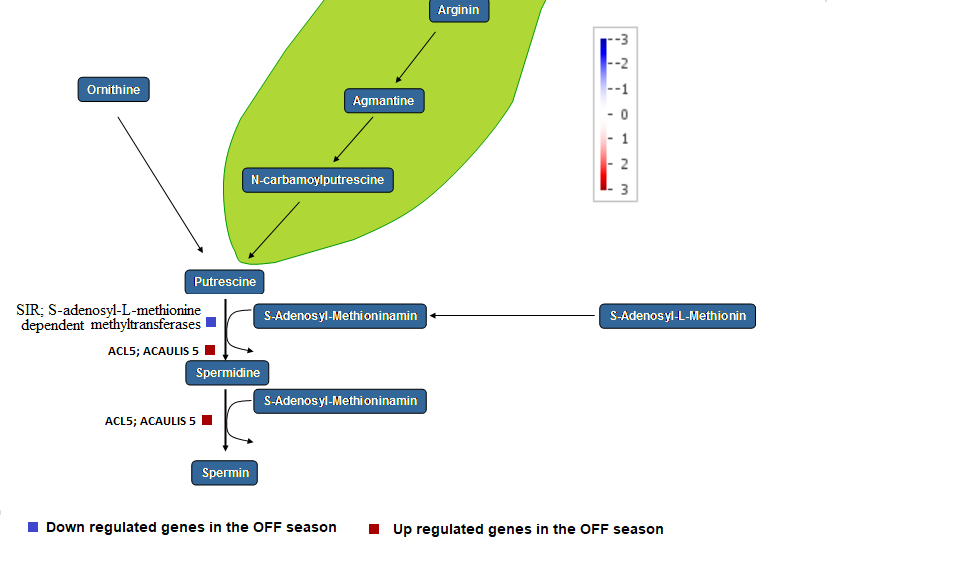

Supplement: Supplementary file 1 [file genes-11-00851-s001.zip › Supplementary_Files/Figure S3.tif]

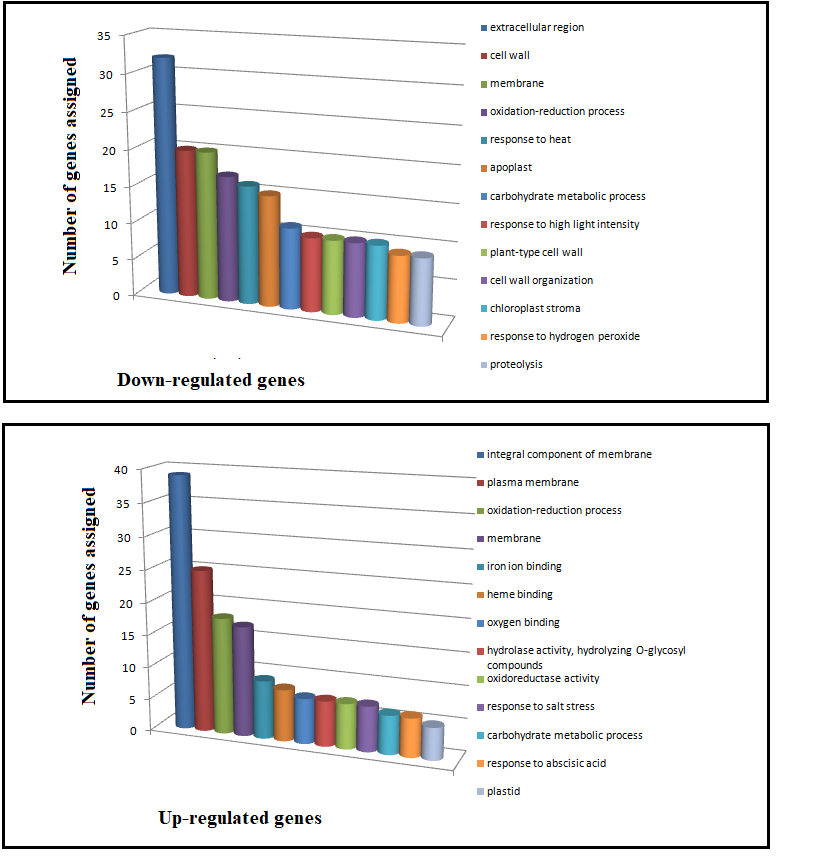

Supplement: Supplementary file 1 [file genes-11-00851-s001.zip › Supplementary_Files/Figure S4.tif]

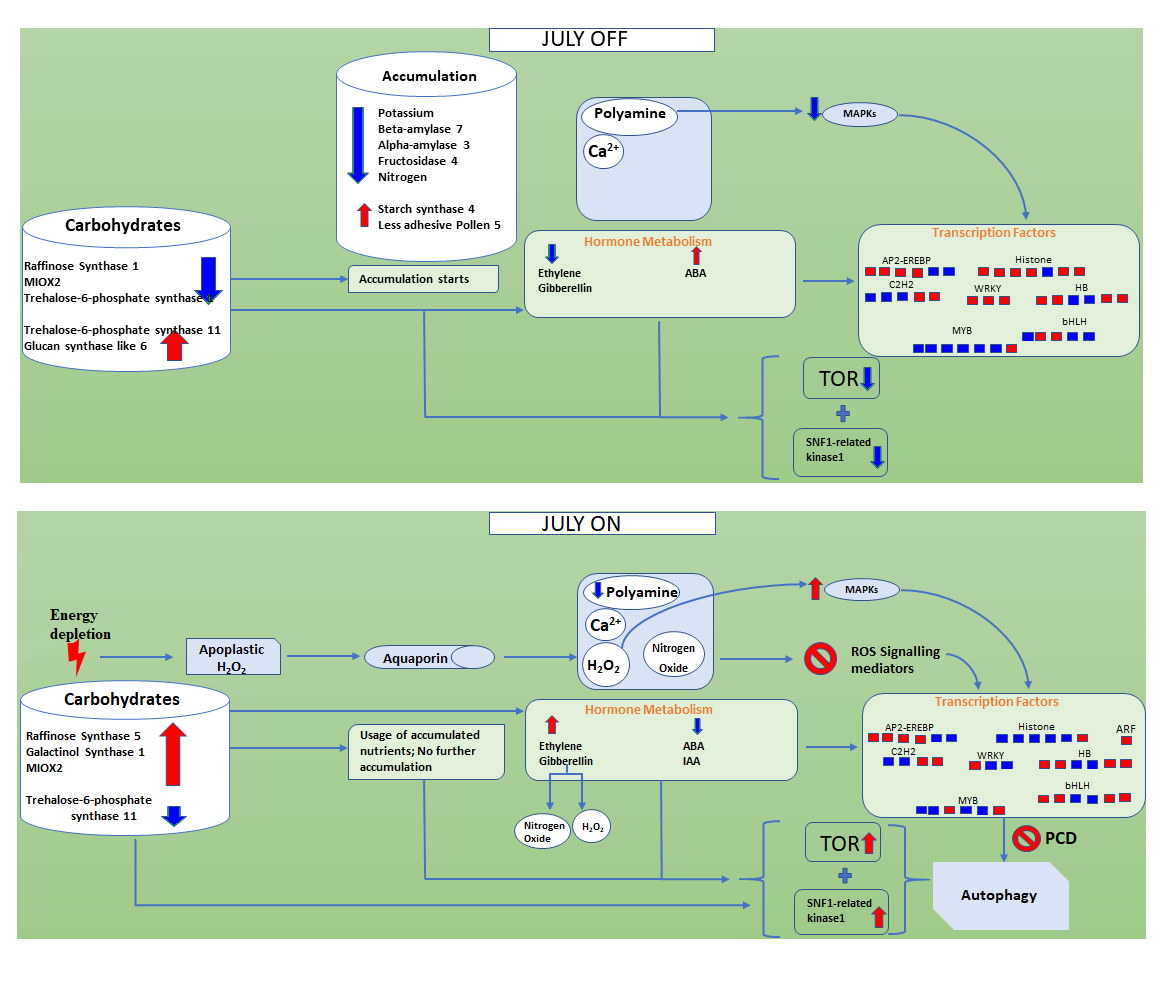

Supplement: Supplementary file 1 [file genes-11-00851-s001.zip › Supplementary_Files/Figure S5.tif]

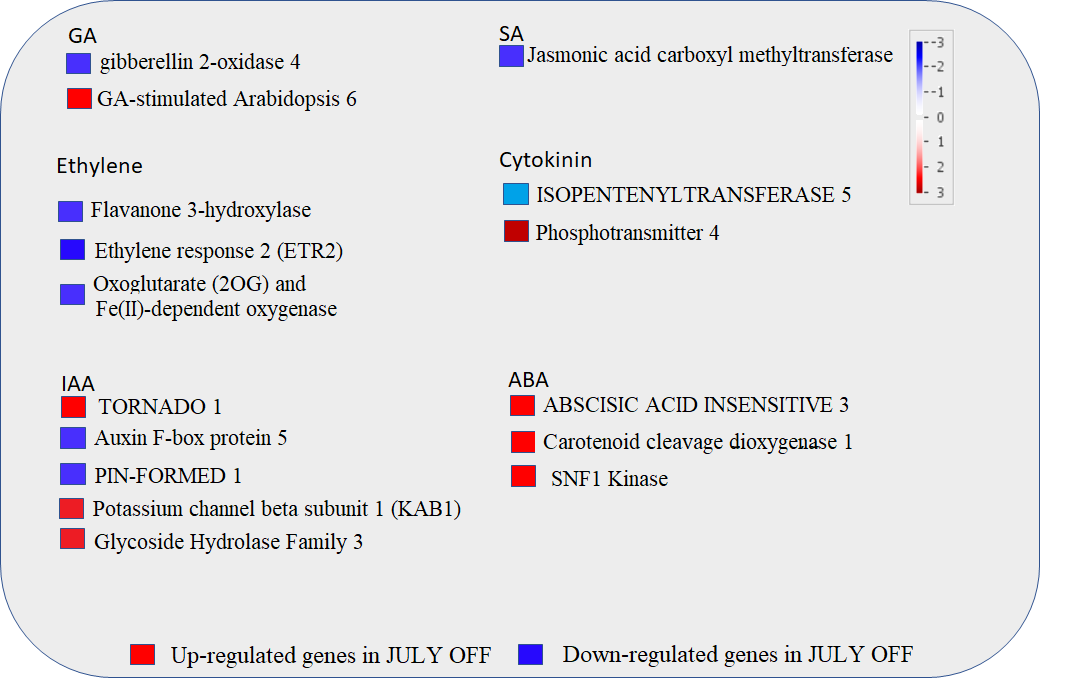

Supplement: Supplementary file 1 [file genes-11-00851-s001.zip › Supplementary_Files/Figure S6.tif]

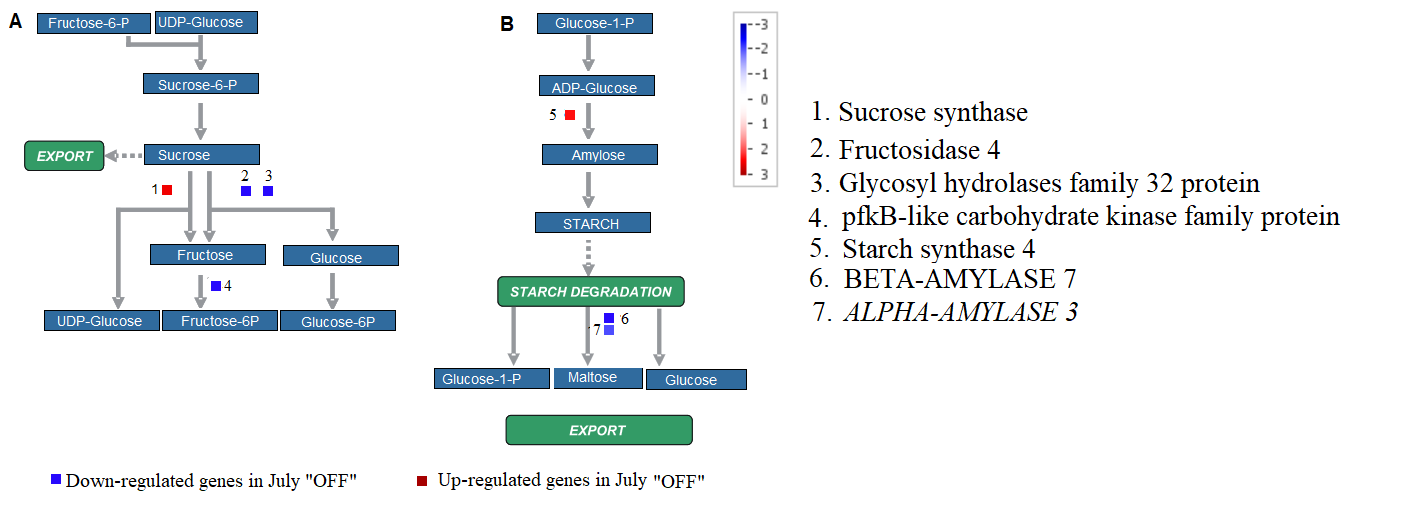

Supplement: Supplementary file 1 [file genes-11-00851-s001.zip › Supplementary_Files/Figure S7.tif]
